# A multiple hypothesis approach to explain species richness patterns in neotropical stream-dweller fish communities

**DOI:** 10.1371/journal.pone.0204114

**Published:** 2018-09-19

**Authors:** Thiago Bernardi Vieira, Carla Simone Pavanelli, Lilian Casatti, Welber Senteio Smith, Evanilde Benedito, Rosana Mazzoni, Jorge Iván Sánchez-Botero, Danielle Sequeira Garcez, Sergio Maia Queiroz Lima, Paulo Santos Pompeu, Carlos Sérgio Agostinho, Luciano Fogaça de Assis Montag, Jansen Zuanon, Pedro De Podestà Uchôa de Aquino, Mauricio Cetra, Francisco Leonardo Tejerina-Garro, Luiz Fernando Duboc, Ruanny Casarim Corrêa, María Angélica Pérez-Mayorga, Gabriel Lourenço Brejão, Nadayca Thayane Bonani Mateussi, Míriam Aparecida de Castro, Rafael Pereira Leitão, Fernando Pereira de Mendonça, Leandra Rose Palheta da Silva, Renata Frederico, Paulo De Marco

**Affiliations:** 1 Laboratório de Ictiologia de Altamira, Universidade Federal do Pará (UFPA), Altamira, Para, Brasil; 2 Núcleo de Pesquisas em Limnologia, Ictiologia e Aqüicultura (Nupelia), Universidade Estadual de Maringá (UEM), Maringá, Paraná, Brasil; 3 Laboratório de Ictiologia, Universidade Estadual Paulista (UNESP), São José do Rio Preto, São Paulo, Brasil; 4 Universidade Paulista, Sorocaba, São Paulo, Brasil; 5 Departamento de Ecologia, Universidade do Estado do Rio de Janeiro (UERJ), Rio de Janeiro, Rio de Janeiro, Brasil; 6 Departamento de Biologia, Centro de Ciências, Universidade Federal do Ceará (UFC), Fortaleza, Ceará, Brasil; 7 Instituto de Ciências do Mar - LABOMAR, Universidade Federal do Ceará (UFC), Fortaleza, Ceara, Brasil; 8 Departamento de Botânica, Ecologia e Zoologia, Centro de Biociências, Universidade Federal do Rio Grande do Norte (UFRN), Natal, Rio Grande no Norte, Brasil; 9 Departamento de Biologia, Universidade Federal de Lavras (UFLA), Lavras, Minas Gerais, Brasil; 10 Universidade Federal do Tocantins (UFT), Porto Nacional, Tocantins, Brasil; 11 Instituto de Ciências Biológicas, Universidade Federal do Para (UFPA), Belém, Para, Brasil; 12 Coordenação de Biodiversidade, Instituto Nacional de Pesquisas da Amazônia (INPA), Manaus, Amazonas, Brasil; 13 Departamento de Zoologia, Universidade de Brasília, Brasília, Distrito Federal, Brasil; 14 Universidade Federal de São Carlos, Sorocaba, São Paulo, Brasil; 15 Programa de Mestrado em Sociedade, Tecnologia e Meio Ambiente, UNIEvangélica, Anápolis, Goiás Centro de Biologia Aquática, Pontifícia Universidade Católica de Goiás, Goiânia, Goiás, Brasil; 16 Departamento de Ciências Agrárias e Biológicas, Centro Universitário Norte do Espírito Santo (CEUNES), Universidade Federal do Espírito Santo (UFES), São Mateus, Espirito Santo, Brasil; 17 Instituto de Biociências, Universidade Estadual Paulista, Botucatu, São Paulo, Brasil; 18 Departamento de Genética, Ecologia e Evolução, Instituto de Ciências Biológicas, Universidade Federal de Minas Gerais (UFMG), Belo Horizonte, Minas Gerais, Brasil; 19 Departamento de Ecologia, Instituto de Ciências Biológicas, Universidade Federal de Goiás Campus II (UFG), Goiânia, Goiás, Brasil; Universidade Regional Integrada do Alto Uruguai e das Missoes, BRAZIL

## Abstract

Several hypotheses are used to explain species richness patterns. Some of them (e.g. species-area, species-energy, environment-energy, water-energy, terrestrial primary productivity, environmental spatial heterogeneity, and climatic heterogeneity) are known to explain species richness patterns of terrestrial organisms, especially when they are combined. For aquatic organisms, however, it is unclear if these hypotheses can be useful to explain for these purposes. Therefore, we used a selection model approach to assess the predictive capacity of such hypotheses, and to determine which of them (combined or not) would be the most appropriate to explain the fish species distribution in small Brazilian streams. We perform the Akaike’s information criteria for models selections and the eigenvector analysis to control the special autocorrelation. The spatial structure was equal to 0.453, Moran’s I, and require 11 spatial filters. All models were significant and had adjustments ranging from 0.370 to 0.416 with strong spatial component (ranging from 0.226 to 0.369) and low adjustments for environmental data (ranging from 0.001 to 0.119) We obtained two groups of hypothesis are able to explain the richness pattern (1) water-energy, temporal productivity-heterogeneity (AIC = 4498.800) and (2) water-energy, temporal productivity-heterogeneity and area (AIC = 4500.400). We conclude that the fish richness patterns in small Brazilian streams are better explained by a combination of Water-Energy + Productivity + Temporal Heterogeneity hypotheses and not by just one.

## Introduction

Studies on the distribution pattern of species richness have been of great interest in environmental, biogeographical, and paleontological research programs since the early 19^th^ century [1–3]. However, there is no clear or unique answer to this topic [2,4]. Different biological groups, such as plants [4,5], insects [6,7], amphibians [8], reptiles [9,10], birds [11], and fishes [2,7,12–15], have already been tested for richness patterns. In most cases, the influence of the latitudinal gradient in species richness is the main aspect examined [16], which is considered the oldest manner to explain richness distribution [17]. The pattern usually observed is the increasing of species richness toward the equator, which can be explained by various hypotheses [2,17,18]. However, the decreasing of species richness toward the poles is dependent on the scale and the organism studied [17].

Various hypotheses are used to explain richness distribution, the species-area and species-energy hypotheses being the ones that have stood out [13]. The species-area relationship [19] predicts that species richness increases as a function of the increase in the area, which is a power function. On the other hand, the species-energy hypothesis [20] predicts that species richness is a function of the amount of energy available in the system. A third hypothesis used to explain the richness gradient is environment-energy [21], which is a derivation of the energy-species hypothesis and predicts that there is a direct relationship between the temperature of the environment and species richness. The water-energy hypothesis [22] predicts the species richness as a function of the amount of water and evapotranspiration available in the system. By this way, richness would exhibit greater relationship with the amount of water available in the system at low latitudes, since energy would not be a limiting factor. On the other hand, this relationship would be reversed at high latitudes, with the energy being the limiting factor [1,4,6]. The hypothesis of terrestrial primary productivity [23] predicts that species richness would be limited by terrestrial primary productivity. However, for aquatic environments, this relationship is not so simple, since the aquatic primary productivity tends to be 90 times smaller than terrestrial productivity, and temperate continental aquatic environments are ten times less productive than continental tropical waters [13,14,24,25]. The hypothesis of environmental spatial heterogeneity [26] predicts that areas with greater variation in physical environmental characteristics (greater number of habitats potentially available) would also have greater species richness, since these areas could support more species in one place. Finally, the temporal climatic heterogeneity hypothesis [27] predicts that areas with greater variation of climatic characteristics would have greater richness, because they would support more species over time.

When we assess spatially structured data, such as species richness [28–32], we increase the likelihood of occurrence of type I error, i.e., discarding the null hypothesis when it is actually true [33–35]. In addition, the spatial structuring of data may modify the relationship between the dependent and independent variables, exhibiting a negative effect when it is positive, positive when it is negative, or even a null effect when in fact it is not null [30,32]. Still, many studies that explain the richness gradient of the ichthyofauna are not concerned with this effect. Numerous studies do not use any form of control or integration of spatial structure in the analyses [2,13,36,37].

There are studies that have described the richness gradient in rivers as a function of terrestrial primary productivity of the area drained by the basin and flow at the mouth of a river [2,13]. Other studies relate fish species richness in streams to physical variables, such as width, depth, and flow [36,37]. As a theoretical structure for the relationships found, the following hypotheses are listed: (*i*) energy—represented by the terrestrial primary productivity [2,12,13]; (*ii*) species-area—represented by the area drained by the basin [12]; (*iii*) passive dispersal—represented by the flow [36]; (*iv*) environmental spatial heterogeneity—represented by width and depth; and (*v*) climatic heterogeneity—represented by temperature variation in the environment [37]. However, none of these hypotheses is able to explain richness patterns if they are used isolated, thus requiring the integration of two or more hypotheses [13]. Furthermore, and the way a combination of hypotheses influence the patterns of ichthyofaunal richness is uncertain, since there are no studies that confront systematically the hypotheses and the different combinations of hypotheses with the gradient of fish species richness. Therefore, we sought to select models to assess the predictive capacity of the hypotheses, trying to find which hypotheses or combinations of hypotheses would be the most appropriate to explain the species richness distribution in small Brazilian streams. We tested the following combination of hypotheses: (*i*) energy—fish communities of streams are related to evapotranspiration rates; (*ii*) water-energy—species richness is structured on the basis of evapotranspiration and the average annual rainfall; (*iii*) terrestrial primary productivity—species richness is dependent on the terrestrial primary productivity; (*iv*) temporal heterogeneity—the variation of temperature and annual rainfall is used as predictor variables to determine the species richness of streams; (*v*) area—species richness is related to the amount of available area in the basin; and (*vi*) neutral—the geographical distances between the collection sites determinate the species richness of streams.

## Materials and methods

### Database

**Fig 1:**
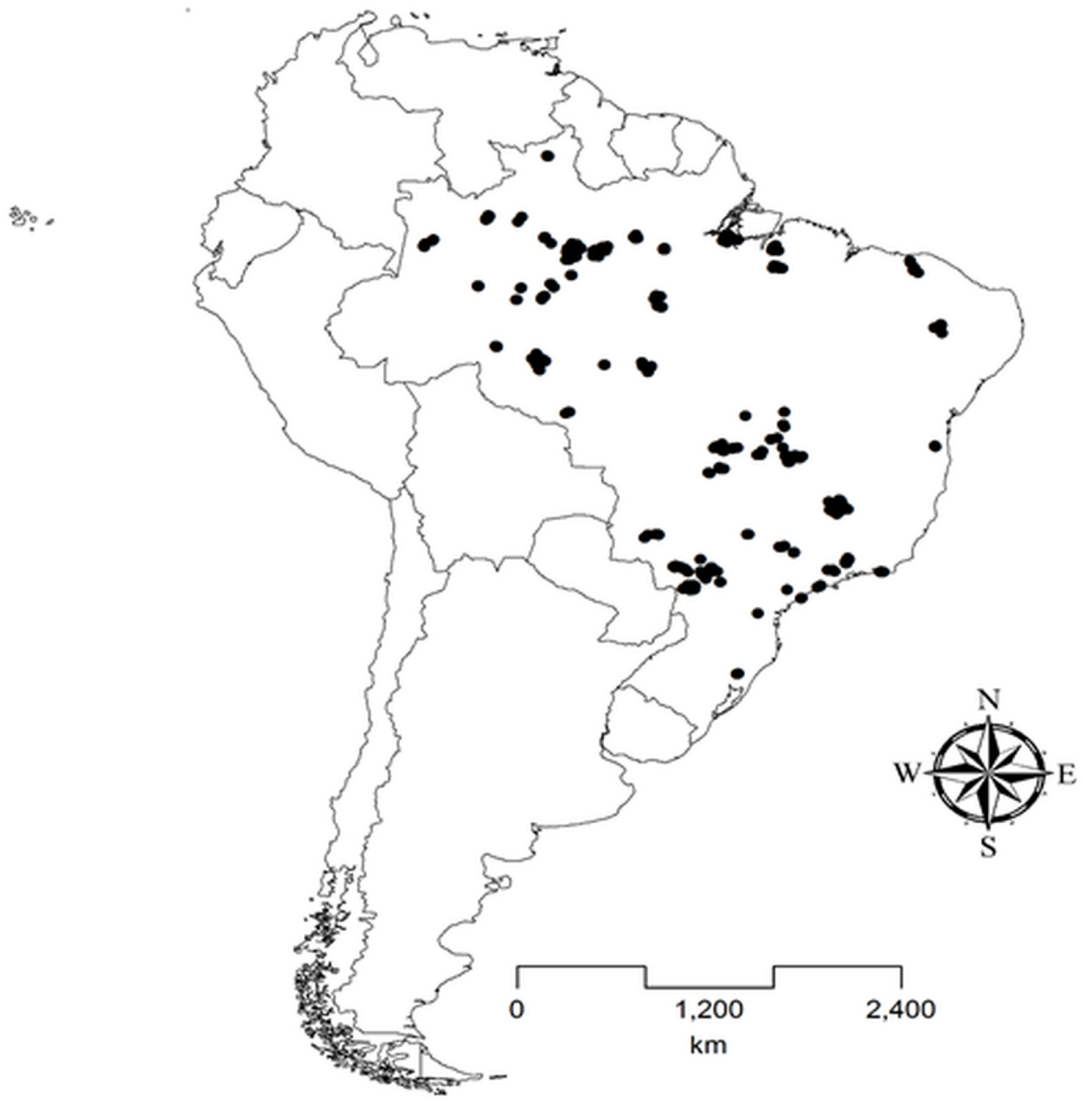
Spatial location of the streams assessed (black dots) in Brazil, South America.

Databases obtained in field collections carried out by the authors were used for testing the hypotheses. These collections should meet the following criteria to be entered in the database: (*i*) collection carried out in first-to third-order streams; (*ii*) georeferenced streams; (*iii*) sampling of ichthyofauna carried out with electric fishing, trawl net, and/or hand net; (*iv*) a minimum of 50-meter stretch of stream sampled by site; (*v*) a single sample site by stream; (*vi*) species identified by experienced researchers in accordance with the taxonomic literature [14,38–52]; (*vii*) list of species per sampled site; and (*viii*) collections carried out in locations with the lowest possible anthropic impact. Scientific articles published in journals, monographs, dissertations, and theses were compiled to supplement the database. CAPES journals website (https://goo.gl/D2gE54) and the keywords ‘peixe’, ‘fish’, ‘riacho’, ‘stream’, ‘lista’, and ‘checklist’ were used for the literature search. In all cases, only the studies that met the criteria previously determined were included in the database. These criteria were chosen in order to ensure comparability between samples and decrease the heterogeneity of the database. At the end of the search, 18 studies that addressed 89 streams were included in the database (S1 Table). The streams from the literature were initially compiled in the database of the collaborators with 570 streams, totaling 659 streams (Fig 1 and S2 Table). Fish species richness was determined for each stream of the database.

### Macroecological variables

For testing our hypotheses, we used the variables that were originally considered by the authors as determinant for the richness gradient. We used the following variables set to test the isolated or combined hypothesis: January (AETJan) and June evapotranspiration (AETJune), with the both months representing respectively the warmest and coldest period along the year; primary productivity (PP); annual temperature variation (TempVar); annual average rainfall (AveRF); annual rainfall variation (ARV); and flow accumulation (FAC) used as a proxy for the area drained within the basin (Table 1; S1 Fig). The use of FAC allows performing faster and easier analyses and, since it has high correlation with the drained area, there is no information loss.

**Table 1:** Descriptive statistics of the variables used in the models. The i-v numbers following each variable indicates in which hypothesis the variable was used: i. Energy, ii. energy-water, iii. terrestrial primary productivity, iv. temporal heterogeneity, v. area. All data are available on the S2 Table.

Variable	Code	Mean	Standard Deviation
Species richness	SR	11.517	9.347
January evapotranspiration (mm day^-1^)^i, ii^	AETJan	100.028	41.490
June evapotranspiration (mm day^-1^)^i, ii^	AETJun	66.735	39.275
Primary productivity (cal. m^2^ day)^iii^	PP	8086.156	3313.487
Annual temperature variation (°C * 10)^iv^	TempVar	1023.135	783.507
Annual average rainfall (mm)^ii, iv^	AveRF	1866.077	563.649
Annual rainfall variation (mm)^iv^	ARV	57.131	22.580
Flow accumulation^v^	FAC	532.294	601.729

The AET and PP are items of MODIS (Moderate Resolution Imaging Spectroradiometer) satellite images with information available from 2000 to 2012 on the website of the Laboratory for Image Processing and Geoprocessing of the Federal University of Goiás—LAPIG UFG (https://goo.gl/F1iWvy). The average rates of these variables were used for the analyses. For the calculation of these values, we obtained the images of AET (available monthly) occurred in January and June, and PP (available annually), both from 2000 to 2012. The images of the different years were added and divided by 12, composing a new image that represents 12-year average value of the variable. This procedure was repeated for AETJan, AETJune, and PP data. TempVar, AveRF, and ARV were retrieved from the IPCC climate scenario: A1, available at WORLDCLIM (http://www.worldclim.org). These variables result from interpolation models built with data collected from 1950 to 2000 by the Global Historical Climate Network Dataset (GHCN).

FAC data were retrieved from Hydro-1k digital elevation global model (http://edcdaac.usgs.gov/gtopo30/hydro/). FAC are products from digital elevation models of the GTOPO30 project developed by US Geological Survey-EROS. Since the resolution (pixel size) of all the images were originally 1 x 1 km, they were adjusted to 15 x 15 km. This way, the information for each site was an average value, product of 225 pixels, and not just the value of one pixel.

### Statistical analyses

In all analyses we perform General Linear Model as statistical framework. For testing the energy hypothesis, a model between SR and AETJan and AETJune was built in order to include the energy input during the warmest and the coldest periods of the year. For testing the water-energy hypothesis, a model between SR and AETJan, AETJune, and AveRF was built, since the evapotranspiration represent the energy input into the system and the AveRF represents the annual water availability. For the productivity hypothesis, the SR was related to the PP, which represents the annual terrestrial primary productivity of the site. The hypothesis of temporal heterogeneity was tested using the ARV and TempVar variables, because these variables represent the annual rainfall and temperature variation. For the area hypothesis, species richness was related to the FAC. Finally, a model between SR and the geographical distance of the sites was built for testing the neutral hypothesis. All the models considered (Table 2) were least squares linear models and the geographical distance used was the spatial eigenvectors mapping [53].

**Table 2:** Variables used to explain fish species richness in streams.

Hypothesis	Variables included in the model
Energy	AETJan + AETJune
Water-Energy	AETJan + AETJune + ARV
Terrestrial Productivity	PP
Temporal Heterogeneity	TempVar + ARV
Area	FAC
Neutral	Spatial filters

ETJan: January evapotranspiration; ETJune: June evapotranspiration; PP: primary productivity; TempVar: annual temperature variation; AveRF: annual average rainfall; ARV: annual rainfall variation; and FAC: flow accumulation

**Fig 2:**
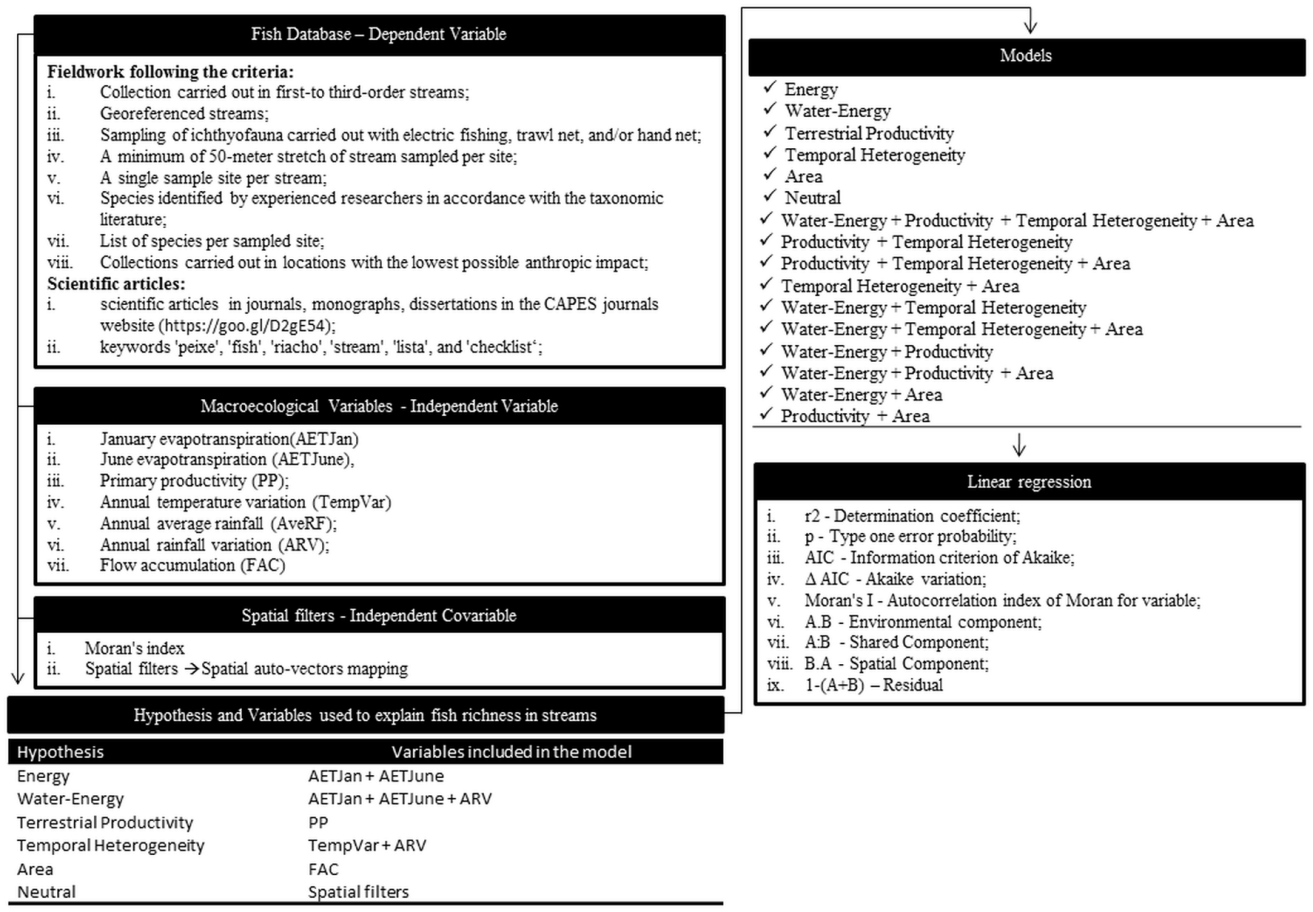
Flowchart representing the statistical procedure used in the article.

We used Moran’s index (Moran’s I) to identify the autocorrelation in the species richness. Spatial auto-vectors mapping (spatial filters) was used to control the spatial structure [53], considered the best way to control the spatial structure [32]. The spatial structure of fish species richness was equal to 0.453 according to Moran’s I, requiring 11 spatial filters to control the autocorrelation effect (S2 Fig). The first spatial filter (SF1) shows the highest values for the streams located in the upper portion of the Amazon region. The second filter (SF2) shows the highest values located in the northern portion of the Paraná region. The fourth filter (SF4) shows high values for the streams of the southernmost portion of the Amazon region and the western Paraná region. The other filters do not show a clear pattern (S2 Fig). All the spatial filters were incorporated into the models as covariates (Table 2), except in the neutral hypothesis, in which the filters were used as predictor variables. Moran’s I value and Akaike’s information criterion (AIC) were presented for all models, which were compared through the AIC variation. Since some studies have already found that a single hypothesis cannot explain variation in fish species richness [12,13], we also tested the combinations between the water-energy, terrestrial productivity, temporal heterogeneity, and area models. To avoid repetition of variables in the tests, the energy and neutral models were removed from the combinations, since the variables that represent the energy model are already included in the water-energy model, and the neutral model is already represented by spatial filters in the other models. In this way, the hypotheses combined were two, three, and four at a time. Spatial auto-vectors mapping and regression analyses were carried out using the SAM software—Spatial Analysis in Macroecology [54,55]. Collinearity between the variables was measured through the variance inflation factor (VIF), which quantifies the multicollinearity of predictor variables (Fig 2). This index ranges from 1 (no collinearity) to positive infinity and provides an estimate of how much the variance of a regression coefficient is increased by collinearity. VIF values less than 10 are considered acceptable. After testing the hypotheses, species richness was subjected to a multiple regression analysis, without the use of special filters and using only the significant variables. The model obtained with this analysis was spatialized, producing a map of fish species richness in streams. For testing the accuracy of the model, we extracted the values predicted for each stream present in the analyses and subtracted the value of the richness observed from the estimated value. Thereby, positive values represent sites overestimated by the model, and negative values represent those underestimated by the model. With these values, we performed a regression analysis between the predicted and observed species richness, using all sites together and by river basin (Amazon, East Atlantic, Western Northeast Atlantic/Eastern/Parnaíba, South/Southeast Atlantic, Paraguay/Paraná, São Francisco, Tocantins-Araguaia, and Uruguay). The regression model and the map were made using the R environment [56] with Vegan [57] package.

## Results

All the models built to explain the fish species richness of streams were significant and had adjustments ranging from 0.301 to 0.416 (Tables 3 and 4). They showed strong spatial component and shared low adjustments for environmental data (Tables 3 and 4). When the models were tested individually (Table 3), the temporal heterogeneity hypothesis—according to AIC—was the best adjusted for species richness. However, when we tested the hypotheses combined (Table 4), the models built by the hypotheses "water-energy, productivity, and temporal heterogeneity" and "water-energy, productivity, temporal heterogeneity, and area" were those that best explained the fish species richness of streams.

**Table 3:** Regression coefficients and comparison between the five hypotheses used to explain the distribution of fish species richness in streams.

Hypotheses	*r*^2^	*p*	AIC	Δ AIC	Moran’s I	*r*^2^ for partial regression results			
						A.B	A:B	B.A	1-(A+B)
Temporal Heterogeneity	0.401	<0.001	4510.500	0.000	0.093	0.031	0.054	0.316	0.599
Energy	0.376	<0.001	4535.200	24.700	0.115	0.007	0.090	0.279	0.624
Area	0.301	<0.001	4538.400	27.900	0.122	0.005	0.053	0.316	0.626
Water-Energy	0.376	<0.001	4538.900	28.400	0.114	0.007	0.143	0.226	0.624
Neutral	0.369	<0.001	4539.900	29.400	0.128	-	-	-	0.631
Terrestrial Productivity	0.370	<0.001	4541.600	31.100	0.131	0.001	0.001	0.370	0.628

r^2^—Coefficient of determination; p—Type one error probability; AIC—Information criterion of Akaike; Δ AIC—Akaike variation; Moran’s I—Autocorrelation index of Moran for variable; A.B—Environmental component; A:B—Shared Component; B.A—Spatial Component; 1-(A+B)–Residual

**Table 4:** Regression coefficients and comparison between the combinations of hypotheses to explain the distribution of fish species richness in streams. AIC: Akaike’s information criterion.

Combination of hypotheses	*r*^2^	*p*	AIC	Δ AIC	Moran’s I	*r*^2^ for partial regression results			
						A.B	A:B	B.A	1-(A+B)
Water-Energy + Productivity + Temporal Heterogeneity	0.416	<0.001	4498.800	0.000	0.096	0.046	0.121	0.249	0.584
Water-Energy + Productivity + Temporal Heterogeneity + Area	0.416	<0.001	4500.400	1.700	0.096	0.047	0.120	0.249	0.584
Productivity + Temporal Heterogeneity	0.407	<0.001	4502.100	3.400	0.097	0.038	0.057	0.312	0.593
Productivity + Temporal Heterogeneity + Area	0.408	<0.001	4503.700	4.900	0.097	0.038	0.057	0.312	0.592
Temporal Heterogeneity + Area	0.400	<0.001	4510.400	11.600	0.097	0.030	0.055	0.315	0.600
Water-Energy + Temporal Heterogeneity	0.400	<0.001	4514.200	15.400	0.095	0.031	0.133	0.237	0.600
Water-Energy + Temporal Heterogeneity + Area	0.401	<0.001	4515.500	16.800	0.095	0.031	0.132	0.237	0.599
Water-Energy + Productivity	0.384	<0.001	4530.100	31.300	0.119	0.014	0.144	0.226	0.616
Water-Energy + Productivity + Area	0.384	<0.001	4531.800	33.000	0.119	0.014	0.144	0.226	0.616
Water-Energy + Area	0.376	<0.001	4537.600	38.900	0.115	0.007	0.143	0.226	0.624
Productivity + Area	0.370	<0.001	4540.400	41.600	0.132	0.001	0.001	0.369	0.630

r^2^—Coefficient of determination; p—Type one error probability; AIC—Information criteria of Akaike; Δ AIC—Akaike variation; Moran’s I—Autocorrelation index of Moran for variable; A.B—Environmental component; A:B—Shared Component; B.A—Spatial Component; 1-(A+B)—Residual

**Fig 3:**
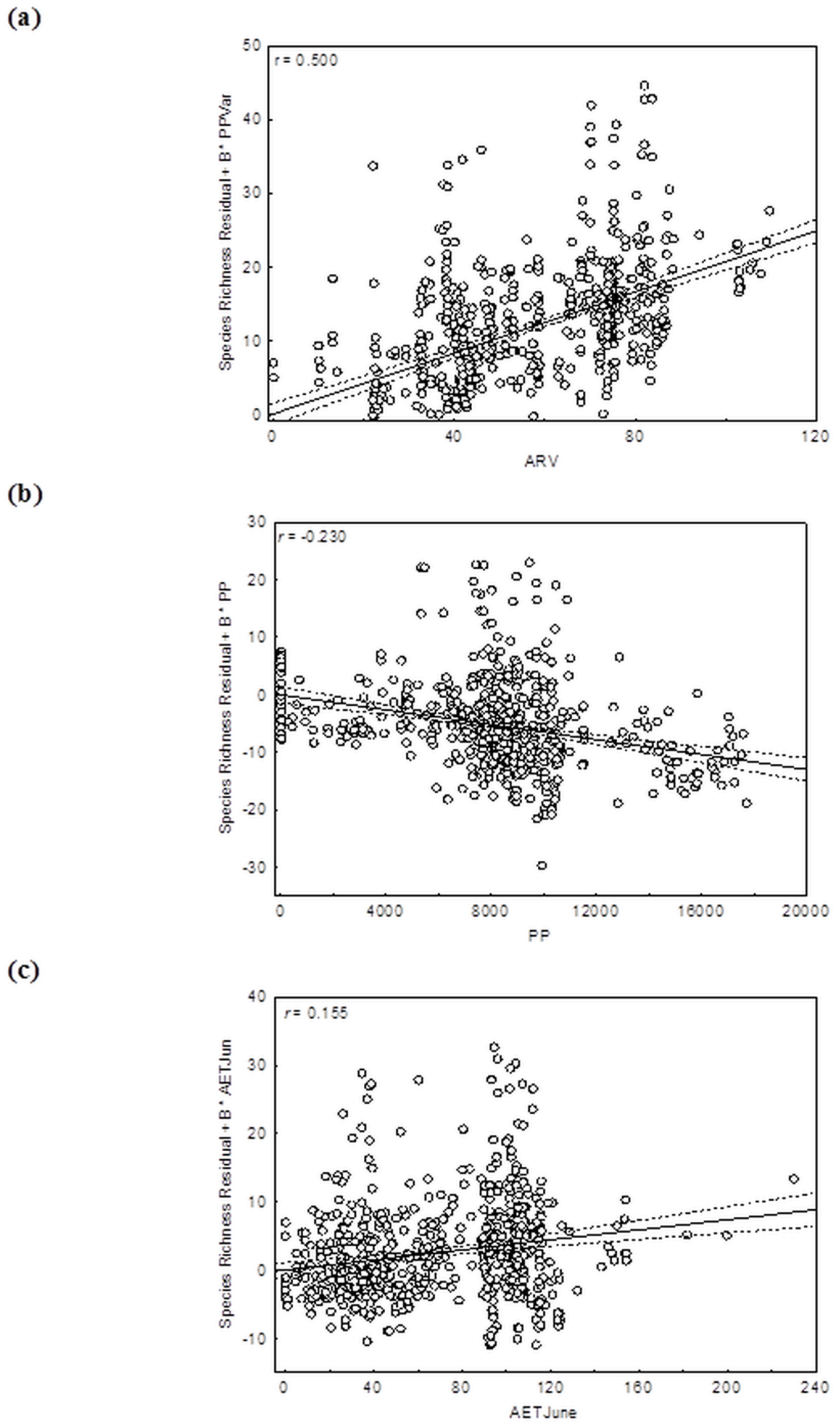
Partial regression between the predictor variables and fish species richness of streams. Species richness as a function of a) the annual rainfall variation (ARV); b) the terrestrial primary productivity (PP) and c) June evapotranspiration (AETJune).

The models built with the combinations of hypotheses were better adjusted for the richness distribution than the individual models, since the Δ AIC between the best individual model (AIC = 4510.500) and the best combined model (AIC = 4498.800) was 11.700. The best combination of hypotheses was "water-energy, productivity, and temporal heterogeneity", with 0.416 adjustment and 0.096 spatial autocorrelation for the residues (Table 4), showing the fish species richness as a function of annual rainfall variation, primary productivity, and June evapotranspiration (Table 5; Fig 3). Actually, two combinations of models had the same explanation power to species richness i) Water-Energy + Productivity + Temporal Heterogeneity and ii) Water-Energy + Productivity + Temporal Heterogeneity + Area. However, the Δ AIC between these two sets was less them two, indicate the same explanation power of these. So the choice of the set with fewer variables (Water-Energy + Productivity + Temporal Heterogeneity) is more parsimonious.

**Table 5:** Results of the regression analyses using the best set of models (Water-Energy + Productivity + Temporal Heterogeneity) as predictors of fish species richness, and spatial filters as covariates.

Variable	SC	VIF	*t*	*p*
Constant	0.000	0.000	0.275	0.784
AETJan	-0.069	2.172	-1.560	0.119
AETJune	0.155	5.634	2.159	**0.031**
AveRF	0.109	5.539	1.539	0.124
PP	-0.230	3.375	-4.148	**<0.001**
TempVar	<0.001	8.739	-0.007	0.995
ARV	0.500	7.788	5.934	**<0.001**
SF1	0.376	10.745	3.803	**<0.001**
SF2	-0.342	6.007	-4.620	**<0.001**
SF3	-0.134	1.864	-3.255	**0.001**
SF4	0.328	1.219	9.832	**0.000**
SF5	0.064	2.850	1.250	0.212
SF6	0.180	2.053	4.170	**<0.001**
SF7	0.268	2.065	6.187	**<0.001**
SF8	0.295	1.651	7.597	**<0.001**
SF9	0.280	1.739	7.033	**<0.001**
SF11	0.073	1.241	2.182	**0.029**
SF17	0.205	1.032	6.677	**<0.001**

AETJan: January evapotranspiration; AETJune: June evapotranspiration; PP: primary productivity; TempVar: annual temperature variation; AveRF: annual average rainfall; ARV: annual rainfall variation; SF1-SF17: spatial filters; SC: standard coefficient; VIF: variance inflation factor; p: Type one error probability. Significant P values (P<0.05) are in bold.

Annual rainfall variation had greatest effect on fish species richness, with coefficient 0.500 (Table 5; Fig 3a). June evapotranspiration and primary productivity showed lower effect than Annual rainfall variation. Primary productivity showed negative effect with coefficient -0.230 (Table 5; Fig 3b), and AETJune showed positive coefficient 0.155 (Table 5; Fig 3c). None of the models was affected by the collinearity of predictor variables, since VIF was less than 10 (Table 5).

**Fig 4:**
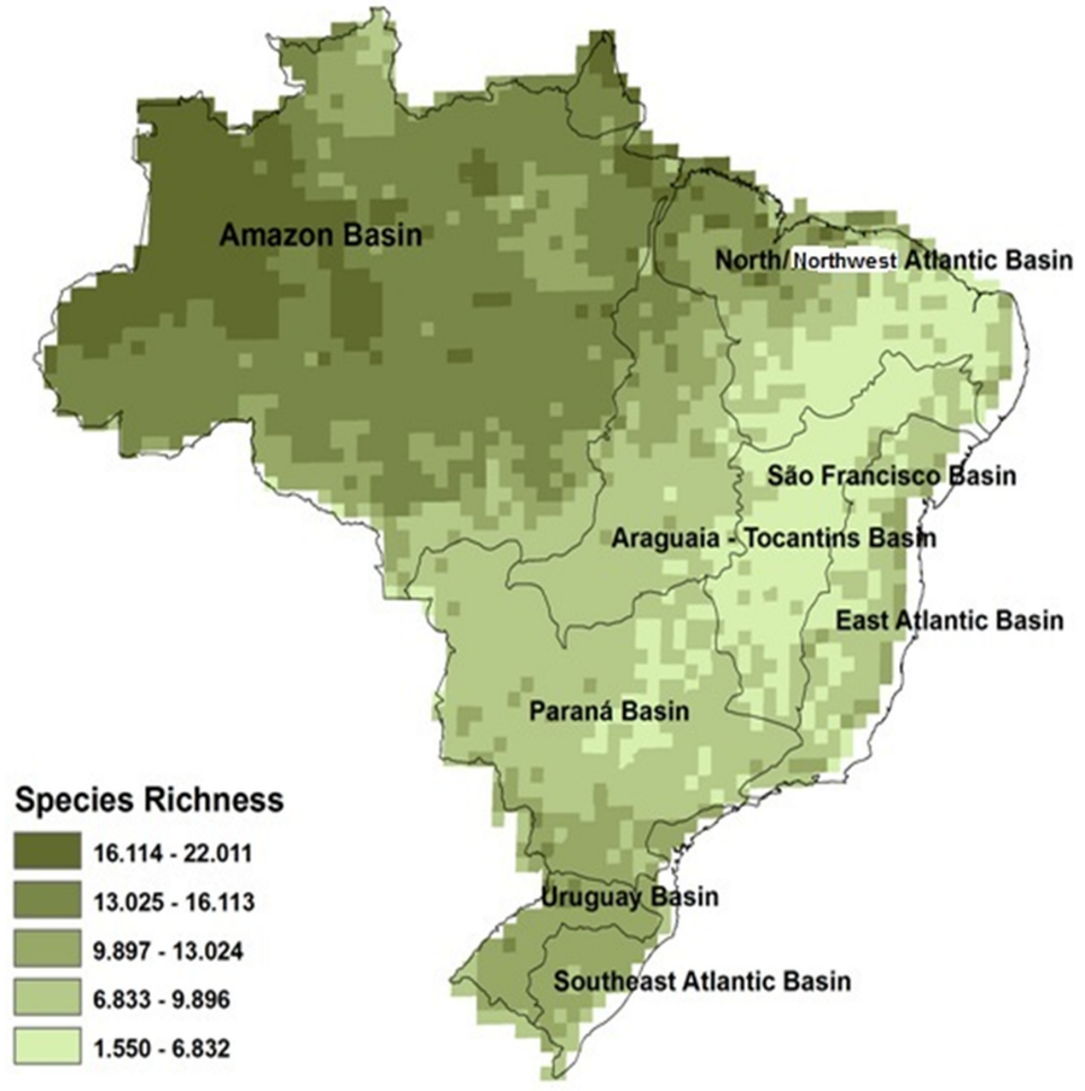
Fish species richness prediction for 1^st^ to 3^rd^ order streams in Brazil. The map was drawn up from the regression model found.

The map of fish species richness for lower than third-order streams shows the richest streams in the northwest portion of the Amazon hydrographic region, with up to 24 fish species, and the poorest streams in the northern region of the São Francisco hydrographic region, with a single species predicted for streams (Fig 4). The southern Paraná hydrographic region, the entire Uruguay hydrographic region, and middle portions of the Tocantins River and North/Northeast Atlantic showed median richness values (Fig 4). The analysis of the model accuracy as a whole shows that there were more underestimated than overestimated sites (S3 Fig). The underestimation of the model reaches up to 40 species, and the overestimation of the model does not exceed 20 species. When each basin was assessed separately, we identified that the Southeast Atlantic Basin showed overestimated sites, reaching approximately 12 species with few underestimated sites two sites showing errors of four species at the most. The Paraná Basin and the East Atlantic Basin showed overestimation of up to 10 species, and the Araguaia-Tocantins Basin showed the lowest overestimated values, with only five species. The highest underestimation values were found in the Amazon Basin, which underestimated the richness up to 40 species, followed by the Paraná Basin and Araguaia-Tocantins Basin with underestimation of 30 species. The São Francisco Basin showed underestimation of 14 species and overestimation of eight species, and the North/Northwest Atlantic Basin had overestimation of eight species and underestimation of up to 12 species (S3 Fig).

## Discussion

When we tested all hypotheses separately, the temporal heterogeneity best explains the distribution of fish species richness in streams. However, under a multiple-hypotheses approach, we observed that the water-energy + productivity + temporal heterogeneity hypotheses together best described the richness distribution. Although it could be considered an acceptable model by AIC, when we add the area (flow accumulation), the results adjustment to this model is not improved. Separately, the flow accumulation variable had no effect on fish species richness. On the other hand, when we confronted the best hypothesis isolated (temporal heterogeneity) with the best set of hypotheses (water-energy, productivity, and temporal heterogeneity), the Δ AIC value is 11.700, arguing that temporal heterogeneity is still the best explanation for the phenomena studied. Multiple hypotheses approach has already been observed in the literature. [13] used the hypotheses energy, area, and history to explain fish species richness in rivers of five continents. In the same paper [13], the authors observed that the hypotheses tested individually were not robust, so they considered them together. [2] used the same data obtained by [12] and [13] and identified the relationship between richness and area and energy (as originally described by [13] and [12]), in addition to the relationship between richness and historical factors. However, none of these studies addressed temporal heterogeneity as an important alternative.

There was a strong relationship between ichthyofauna and space with the spatial filters in all the models assessed, being responsible for more than 50% of the model adjustment. The spatial autocorrelation found in the richness component is a feature repeatedly discussed in the literature [4,6–10,15]. For aquatic organisms, it is expected that the spatial component of the analyses would be greater when organisms are highly dependent on the water, since the hydrological system is longitudinally, laterally, and vertically influenced [37,58]. For fishes, which can disperse only along their own watercourses, a strong spatial component is expected [36], as we found in our study. In spite of the great influence of spatial factors, local conditions cannot be discarded, since they influence the assemblages structure, even in a smaller degree [36,59]. The contribution of the spatial component in the fish species richness distribution is an important factor for the Brazilian ichthyofauna, since, due to historical factors, several sites of endemism that present aggregated distribution in space have been formed [60]. In addition, changes in physical, physicochemical, and chemical properties of watercourses also determine spatial patterns, with gradual changes from the headwaters to the mouth, increasing fish species richness in this same direction [25,61]. There is evidence that this gradient is also related to an increased local heterogeneity [62,63] and the stabilization of hydrological variations [25,58,64]. Other factors that can increase the autocorrelation are the relationship between the area drained, the flow [2,12,13,37,64–67], the terrestrial primary productivity [2,12,13], and the regional richness [12]. The area and the flow are regionally important factors, though of weaker influence at larger scales [2,12,37]. At larger scales, it is expected that the structure of the ichthyofauna is dependent on the colonization and extinction processes, and less influenced by the physical, chemical, and physicochemical processes [36,38,68].

The terrestrial primary productivity was related to richness, but flow accumulation was not reinforcing the idea of climate and topography as strong predictors; however, less predictive at larger scales [2,12,37]. The negative relationship observed between terrestrial primary productivity and fish species richness has also been described by [37] and [2], explaining 76 to 93% of the ichthyofaunal richness variation through the drained area of the basin. [2] and [13] concluded that the increased availability of energy would lead to accumulation of terrestrial biomass, which would be made available to the aquatic environments by being transported laterally. However, terrestrial primary productivity tends to underestimate the aquatic primary productivity by at least 10 times [13,24,25]. When open tropical environments are taken into consideration as is the case of streams of Cerrado this underestimation of the aquatic terrestrial productivity is even greater, since open environments are subject to greater energy input and consequent increase in primary productivity [69]. The input of allochthonous material may occur from the lateral portions of streams and from the upstream portions of the sampled sites, since the organic material can be moved within the body of water in the upstream-downstream direction during the rainy season [70,71]. This way, the little relationship between the terrestrial primary productivity and the ichthyofaunal richness in streams can be attributed both to the underestimation of aquatic primary productivity and the amount of organic matter drifted from upstream sites.

In addition to the terrestrial primary productivity, June evapotranspiration and rainfall variation also demonstrated a positive relationship with the ichthyofaunal richness. The influence of evapotranspiration on diversity patterns is expected only for sites at higher latitudes [1,4,6], with less evident effect at low latitudes or completely replaced by the effect of the amount of water present in the system [4]. The relationship between these two variables (evapotranspiration and water) and fish species richness suggests that the amount of energy would not be a limiting factor at low latitudes, since the available energy is abundant, contrary to the amount of water, which would be the limiting factor. This relationship would be reversed in regions at high latitudes, with the energy being the limiting factor [1,4,6]. This relationship has been found for dragonflies in Europe [6], butterflies in Europe and Africa [29,72], and fishes in this study. [29] and [72] showed that temperature is an important descriptor for Europe, while the water is more important for areas of northern Africa. The water-energy hypothesis was not related to richness. However, in the hypothesis of interaction, a positive relationship between rainfall variation and richness was observed, since this relationship exhibited a larger coefficient than evapotranspiration.

In any large-scale analysis of complex ecological processes, it is expected that the unexplained residues result mostly from the natural complexity of the system and the unmeasured variables, which, combined, can influence the results in several scales. These results suggest that the inclusion of temporal climatic heterogeneity not previously included in similar studies was an important advance of the present study. The predictive map of fish species richness in first-to third-order streams show discrepancies with other studies that presented macroecological models [2,73]. These studies show Brazil as a megadiverse country and richness values over 200 species per basin. However, the model presented here was made only for first to third order streams and not to large rivers and basins. The second caveat is related to the accuracy of the model, which overestimated places, as the Southeast Atlantic Basin, to underestimated places, as the Amazon Basin. This underestimation of richness can be the result of environmental factors that were not included in the study, such as the volume of water, which has been described in the literature as an important predictor of fish species richness in streams [25,38], or a feature of continental freshwater systems, which have non-stationary relationships between the different hydrographic units, highlighting the importance of historical factors of biomes, hydrographic areas, and ecoregions. Anyway, we can conclude that the use of multiple hypothesis approach build better models to explain the fish richness pattern in Brazilian streams.

## Supporting information

S1 Fig Distribution of the variables used for testing the hypotheses. (TIF)

S2 Fig The eleven spatial filters selected to control the spatial autocorrelation of fish species richness in the streams. All the spatial filters are related to positive autocorrelation. The squares are positioned over the streams assessed. The highest values are black squares and the smallest values white squares.
(TIF)

S3 Fig Scatter plots between the estimated fish species richness in streams and the difference between the observed and estimated richness for: (a) all the streams assessed; (b) Southeast Atlantic Basin; (c) Paraná Basin; (d) East Atlantic Basin; (e) Araguaia-Tocantins Basin; (f) Amazon Basin; (g) São Francisco Basin; and (h) North/Northwest Atlantic Basin. (TIF)

S1 Table Bibliographical references and number of streams used in the analyses. (XLSX)

S2 Table Geographic coordinates (decimal degrees) of the streams and variables included in the article. The name and unit of variables are indicating on the Table 1.
(XLSX)
