# Supplementary figures and images for: A multiple hypothesis approach to explain species richness patterns in neotropical stream-dweller fish communities
Source: PLoS One. 2018 Sep 19;13(9):e0204114. doi: 10.1371/journal.pone.0204114 (PMC6145546; doi:10.1371/journal.pone.0204114)

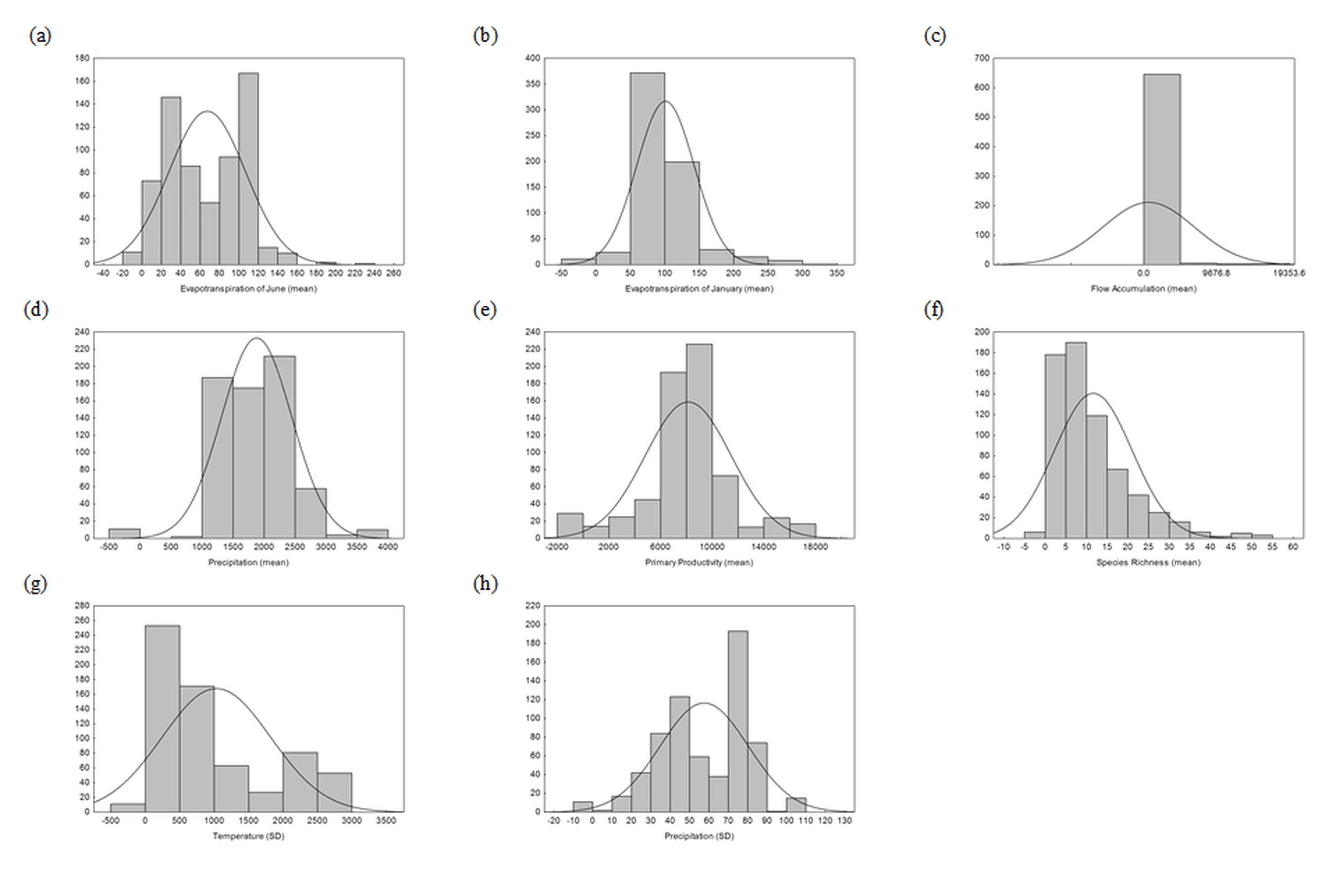

Supplement: S1 Fig — (TIF) [file pone.0204114.s001.tif]

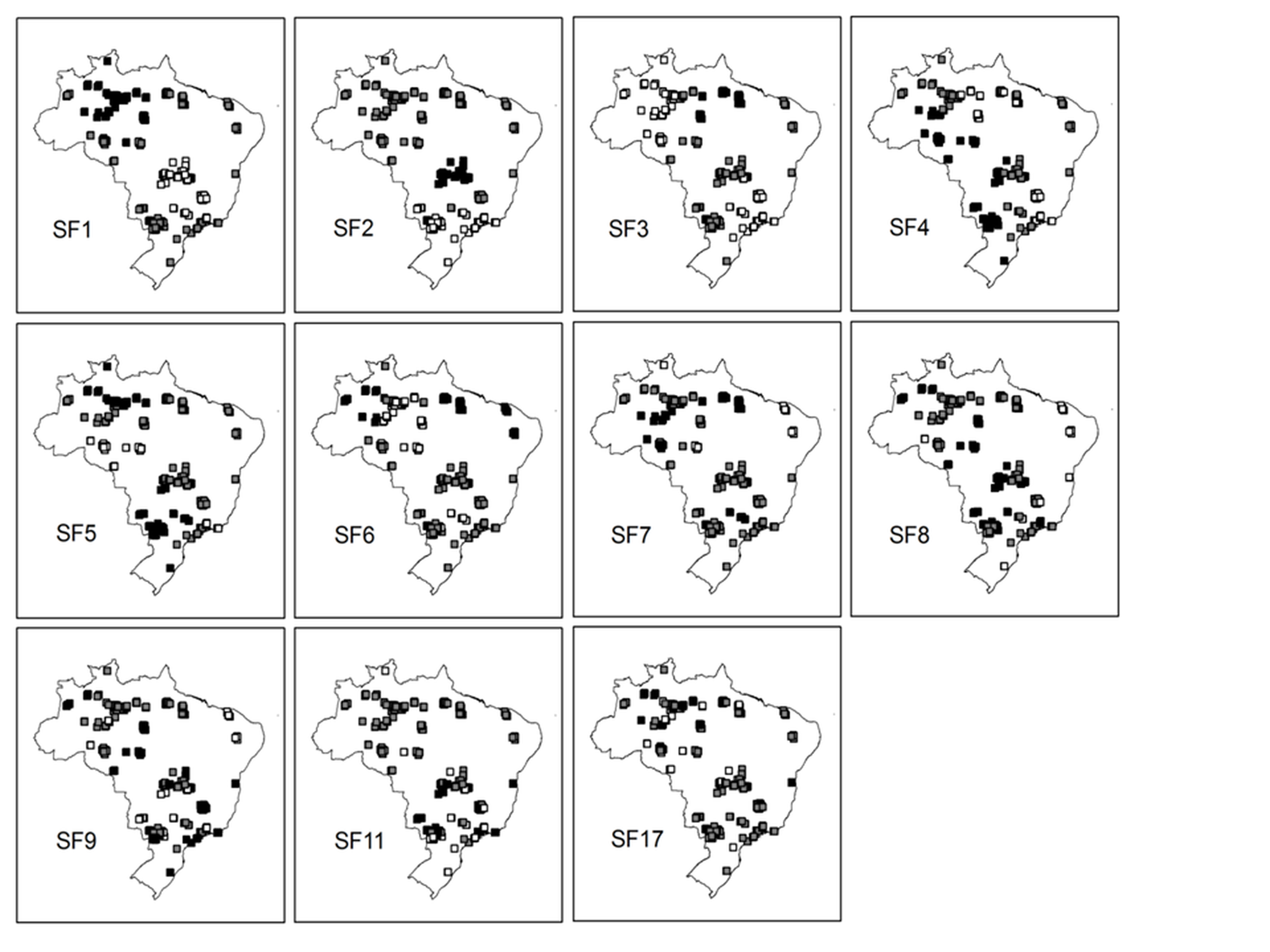

Supplement: S2 Fig — All the spatial filters are related to positive autocorrelation. The squares are positioned over the streams assessed. The highest values are black squares and the smallest values white squares. (TIF) [file pone.0204114.s002.tif]

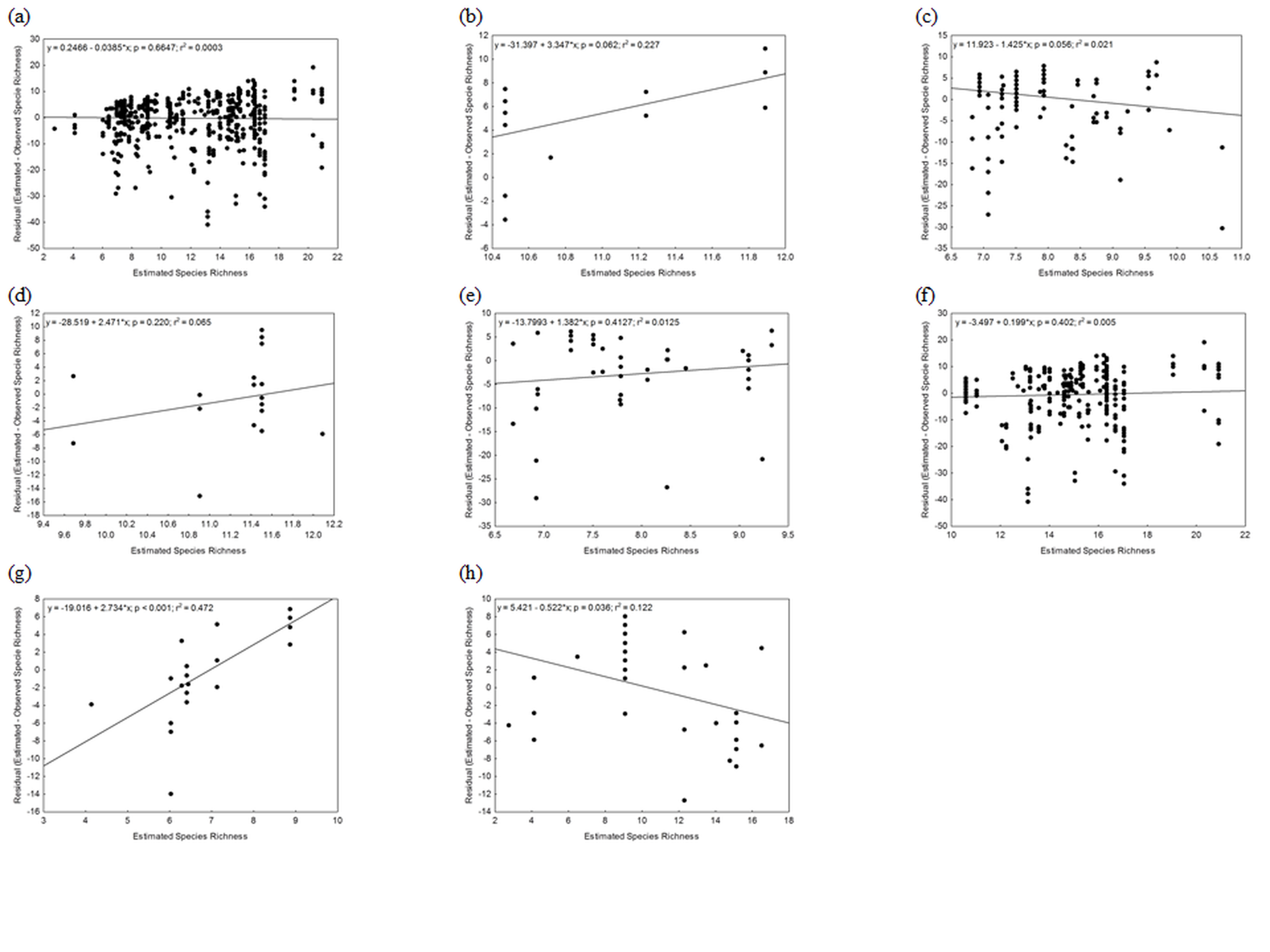

Supplement: S3 Fig — (TIF) [file pone.0204114.s003.tif]
